# Modulation of Hippocampal Astroglial Activity by Synaptamide in Rats with Neuropathic Pain

**DOI:** 10.3390/brainsci11121561

**Published:** 2021-11-26

**Authors:** Igor Manzhulo, Olga Manzhulo, Anna Tyrtyshnaia, Arina Ponomarenko, Sophia Konovalova, Ekaterina Ermolenko, Elena Milkina, Anna Starinets

**Affiliations:** A.V. Zhirmunsky National Scientific Center of Marine Biology, Far Eastern Branch, Russian Academy of Sciences, 690041 Vladivostok, Russia; olga.manzhulo@bk.ru (O.M.); dr.anna.kelvin@gmail.com (A.T.); arina.ponomarenko.93@mail.ru (A.P.); sofanasrew@gmail.com (S.K.); ecrire_711@mail.ru (E.E.); lotlorien27@gmail.com (E.M.); anan.star13@yandex.ru (A.S.)

**Keywords:** N-docosahexaenoylethanolamine, DHEA, synaptamide, chronic constriction injury, neuropathic pain, hippocampus, dentate gyrus, DCX, astroglia, NGF, NMDA

## Abstract

The present study demonstrates that synaptamide (N-docosahexaenoylethanolamine), an endogenous metabolite of docosahexaenoic acid, when administered subcutaneously (4 mg/kg/day, 14 days), exhibits analgesic activity and promotes cognitive recovery in the rat sciatic nerve chronic constriction injury (CCI) model. We analyzed the dynamics of GFAP-positive astroglia and S100β-positive astroglia activity, the expression of nerve growth factor (NGF), and two subunits of the NMDA receptor (NMDAR1 and NMDAR2A) in the hippocampi of the experimental animals. Hippocampal neurogenesis was evaluated by immunohistochemical detection of DCX. Analysis of N-acylethanolamines in plasma and in the brain was performed using the liquid chromatography-mass spectrometry technique. In vitro and in vivo experiments show that synaptamide (1) reduces cold allodynia, (2) improves working memory and locomotor activity, (3) stabilizes neurogenesis and astroglial activity, (4) enhances the expression of NGF and NMDAR1, (5) increases the concentration of Ca^2+^ in astrocytes, and (6) increases the production of N-acylethanolamines. The results of the present study demonstrate that synaptamide affects the activity of hippocampal astroglia, resulting in faster recovery after CCI.

## 1. Introduction

Neuropathic pain is a chronic pain condition resulting from dysfunction of the somatosensory nervous system. Peripheral neuropathic pain can develop after peripheral nerve injury (sensory, motor, or autonomic), which is common in patients with cancer, chronic diabetes, traumatic spinal cord injury, multiple sclerosis, and after certain surgical procedures (thoracotomy, herniorrhaphy, and sternotomy) [1]. The development of neuropathic pain is characterized by the appearance of abnormal sensitivity (dysesthesia), an increased response to pain stimuli (hyperalgesia), and the onset of pain sensitivity in response to non-pain stimuli (allodynia) [2]. In addition, neuropathic pain manifestation can occur in disorders of higher nervous activity, including memory impairment, anxiety, and depression, which indicates the involvement of various brain structures, including the hippocampus, in the pathological processes [3,4]. A number of studies, including our recent research, show that chronic peripheral pain can cause changes in the plasticity of the hippocampus, such as impaired neurogenesis and neuroinflammation. These processes involve glial cells (micro- and astroglia), which are characterized by increased proliferation, hypertrophy, and an increased production of pro-inflammatory cytokines [5,6]. In the course of neuropathic pain development, astroglia is involved in maintaining homeostasis for the central nervous system (CNS) protection. Previously, the important role of astrocytes in the pathogenesis of pain, especially neuropathic pain after peripheral nervous system damage, has been shown. A correlation between astrocyte hypertrophy in various parts of the central nervous system and pain hypersensitivity after damage to peripheral nerves in mice and rats has been observed [7]. In addition, chronic pain is accompanied by astrogliosis, a reactive response of astrocytes characterized by morphological, molecular, and functional changes. In various models of neuropathic pain, astrogliosis is induced at an early stage and persists for a long time (up to 9 months), which indicates the role of this process both in the transition from acute to chronic pain and in the maintenance of pain. It has been previously shown that the inhibition of GFAP expression and astrogliosis after nerve injury correlates with a decrease in the severity of neuropathic pain syndrome [8]. At the same time, reactive astrocytes secrete a large number of signaling molecules, ranging from pro- and anti-inflammatory cytokines to a wide range of growth factors. One of the most important is nerve growth factor (NGF), which plays a key role in the survival and functioning of sensory and sympathetic neurons in the peripheral nervous system, as well as cholinergic neurons in the basal forebrain in the CNS [9].

At the same time, despite the presence of a numerous studies that determine the pathophysiology and mechanisms leading to neuropathic pain, the unsatisfying results in the treatment of patients require additional research in this area. Existing pharmacological drugs, such as antidepressants, non-steroidal anti-inflammatory drugs, anticonvulsants, and opiates, mainly relieve pain symptoms only by nonspecifically decreasing the excitability of neurons, and in some cases, on the contrary, this leads to an even greater worsening of symptoms [10]. Around the world, there is a growing need for alternative pharmacological drugs with effective and safe analgesic properties, lack of toxicity, and proven efficacy in various neuropathologies [11,12]. These properties are possessed by N-docosahexaenoylethanolamine (DHEA), an endogenous metabolite of docosahexaenoic acid (DHA). It was previously shown that an increase in the neuronal DHEA concentration is associated with increased glutamatergic synaptic activity, which is involved in the processes of synaptogenesis. This explains the use of the term “synaptamide” for DHEA. [13]. Previous studies have shown the neurogenic, neuritogenic, and synaptogenic effects of synaptamide in in vitro experiments. In addition, synaptamide inhibits LPS-induced release of proinflammatory production in microglial cells [14,15,16,17]. The anti-inflammatory activity of synaptamide is mediated by the activation of the G-protein coupled receptor GPR110 (ADGRF1), which leads to an increase in cAMP production in neurons [18].

In the present study, we hypothesize that synaptamide has analgesic effects, restores cognitive functions, and improves neurogenesis in the hippocampus after chronic constriction injury (CCI) by modulating astroglial activity and enhancing the expression of NGF and NMDA receptors.

## 2. Materials and Methods

### 2.1. N-Docosahexaenoylethanolamine

The polyunsaturated fatty acids’ concentrate was obtained from the liver of the squid *Berryteuthis magister*, according to the method described by Latyshev et al. (2014) [19]. Further steps for obtaining DHEA with a purity of 99.4% are described in our previous study [17].

### 2.2. Animals

Male Wistar rats (250 ± 20 g, age 3 months) were used for the experiments. The rats were housed 2–4 per cage with ad lib. access to food and water. The rats were maintained at constant temperature (23 ± 2 °C) and humidity (55 ± 15%) with a 12-h light/dark cycle (light at 7:00). All experimental procedures were approved by the Animal Ethics Committee at the A.V. Zhirmunsky National Scientific Center of Marine Biology, Far Eastern Branch, Russian Academy of Sciences (No. 1/2021), according to the international regulations of the European Directive 2010/63/EU and ethical guidelines for the study of experimental pain in conscious animals by the International Association of the Study of Pain.

### 2.3. CCI Procedure and Treatments

To induce neuropathic pain, we used the sciatic nerve chronic constriction injury (CCI) model [20]. The rats were anesthetized with 4.5% isoflurane (Laboratories Karizoo, Barcelona, Spain) in 100% oxygen (VetFlo, Kent Scientific Corporation, Torrington, CT, USA) [21]. After the right sciatic nerve was exposed at mid-thigh level, three ligatures (silk, Ethicon, Somerville, NJ, USA) were placed close to the trifurcation at 2 mm intervals. The ligatures were gradually tightened until a slight twitching of the limb appeared. All animals were randomly divided into 4 groups: “Sham”—sham-operated animals (*n* = 19); “Sham + Syn”—sham-operated rats treated with synaptamide (*n* = 19); “CCI”—vehicle-treated animals with CCI (*n* = 19); “CCI + Syn”—synaptamide-treated rats with CCI (*n* = 19). Groups of sham-operated rats underwent exposure of the sciatic nerve, but without ligation. An antiseptic spray was used to treat the skin after suturing. The animals were immediately injected subcutaneously with synaptamide or vehicle. Ethanol in a concentration not exceeding 5% with the addition of water was used as a carrier for the administration of synaptamide. The total volume of the injected emulsion was 100 μL. A homogeneous, almost transparent emulsion was obtained with constant shaking. Synaptamide emulsion at a dose of 4 mg/kg was administered subcutaneously daily for 14 days after CCI. Animals in the “Sham” and “CCI” groups were treated with a similar volume of vehicle, but without synaptamide.

### 2.4. Behavioral Testing

All behavioral tests were carried out during the light/dark cycle from 7:00 to 19:00. The experimental instruments were cleaned with 70% ethanol thoroughly after testing eacheach animal. A cold plate test was used for cold allodynia assessment (Columbus Instruments, Columbus, OH, USA). For testing, a 30 × 30 cm metal plate with acrylic walls 30 cm high was used. The temperature of the cold plate was +4 °C. The time of the injured limb’s first elevation was recorded and used to assess allodynia. The testing time was 60 s.

Open-field testing was used to assess locomotor activity. The rats were placed in the center of a square Plexiglas arena for 5 min and the number of squares crossed was counted.

The Y-maze was used to assess working memory. The rats were placed in the center of the maze and left for free exploration for 5 min. The criterion for the arm entering was the placement of all 4 paws inside the sleeve. The total number of entries into the arms (N) and the number of consecutive entries into 3 non-repeating arms (M) were evaluated. The spontaneous alternation rate was calculated by the formula: R (%) = M × 100/(N − 2) [22].

An elevated plus-maze (Panlab/Harvard Apparatus, Barcelona, Spain) was used to assess anxiety in animals. The apparatus consisted of two open arms (50 × 14 cm, surrounded by a 1 cm high frame) and two closed arms (50 × 14 cm, surrounded by walls 30 cm high). The animal was placed in the center of the maze with its head towards the open arm, and video was recorded for 5 min using the SMART 3.0 software (Panlab/Harvard Apparatus, Barcelona, Spain). The times spent in the open arms, the closed arms, and the central zone were recorded, with subsequent analysis. The test was carried out on the 13th day of the experiment.

Neuropathic pain and the effect of synaptamide on long-term memory were assessed using the passive avoidance test [23]. The device consisted of light and dark compartments with a sliding door (Panlab/Harvard Apparatus, Barcelona, Spain). When the rat entered the dark compartment, the door was closed, and this was followed by an electrical shock (0.3 mA, 2 s). The experiment was repeated 24 h after the training session but without an electric shock. The latent time of the entry of rats into the dark compartment was measured.

### 2.5. Immunohistochemistry

Brains were harvested 14 days after the surgery. Rats (*n* = 7) were anesthetized with 4.5% isofluorane and perfused with 20 mL of saline followed by 20 mL of cold 4% para-formaldehyde cooked on a 0.1 M phosphate buffer (PBS), pH 7.2. Then, the hippocampus was fixed for 24 h at +4 °C in fresh buffered 4% paraformaldehyde. Further, tissue was processed and embedded in paraffin. Sections (7 μm) were obtained from the ipsi- and contralateral hippocampus. After dewaxing, incubation with primary rabbit antibodies S100β, 1:1000 (ab41548, Abcam, Cambridge, UK), DCX, 1:500 (ab18723, Abcam, Cambridge, UK), and mouse antibodies GFAP, 1:2000 (AMAb91033, Sigma Aldrich, Saint Louis, MN, USA) was carried out overnight at +4 °C. A negative control (without primary antibodies) was performed. Secondary antibodies (PI-1000, anti-rabbit; PI-2000, anti-mouse, Vector Laboratories, Burlingame, CA, USA) were used according to the attached instructions. After washing, preparations were treated with chromogen (Nova RED substrate kit, SK-4800, Vector Laboratories, Burlingame, CA, USA) for 5–10 min. Then sections were washed, dehydrated, and mounted onto slides using a mounting medium (CS705, Dako, Denver, CO, USA).

**Figure 1:**
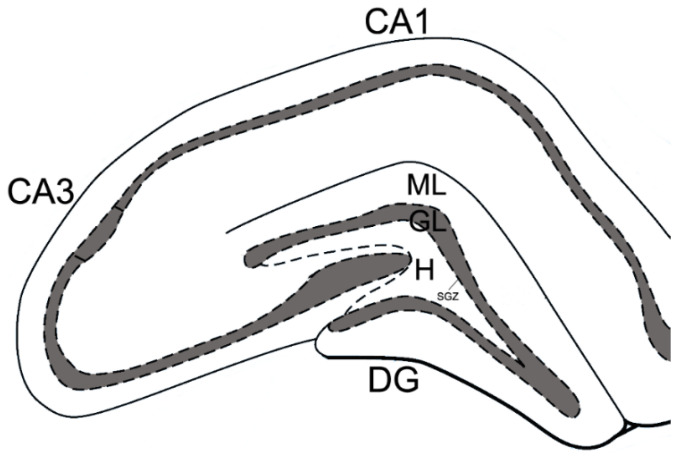
Diagram of the rat hippocampus. Areas of the dentate gyrus (DG): SGZ—subgranular zone, ML—molecular layer of dentate gyrus, GL—granule cell layer, H—hilus of dentate gyrus.

Images were acquired on a microscope (Axio Image Z2, Carl Zeiss, Oberkochen, Germany). For analysis, at least 56 images of the ipsi- and contralateral hippocampus were used for each group of animals. The staining area of the GFAP- and S100β-positive astroglia was determined in each 10th section of the brain. The ratio of the immunopositive staining area to the dentate gyrus area (molecular layer of dentate gyrus, granule cell layer, hilus of dentate gyrus) was expressed as a percentage (Figure 1). The number of DCX-positive neurons/mm^3^ was calculated relative to the area of the dentate gyrus subgranular zone (DG SGZ) of the hippocampus. All measurements were performed by an operator blinded to the sections’ identity using ImageJ software (NIH, Bethesda, MD, USA).

### 2.6. Cell Culture

The primary astrocyte culture was obtained from 1–2 day-old rat pups according to the protocol by Schildge et al. (2013) [24]. To confirm the purity of the astroglial cell line, immunocytochemical analysis was performed using anti-GFAP antibodies (1:2000, Sigma Aldrich AMAB91033, Saint Louis, MN, USA) and S100β (1:2000, Abcam ab868, Cambridge, UK).

Before the experiment, synaptamide was dissolved in 96% ethanol at a concentration of 10 mg/mL. The resulting stock solution was diluted with culture medium, so that the final concentration of ethyl alcohol did not exceed 0.1%. Cells incubated without synaptamide but with vehicle were used as negative controls. For the ELISA assay, astroglia cells were plated in 24-well plates (1 × 10^5^ cells/well) and incubated with 5% CO_2_ at 37 °C. The astrocyte culture was then treated with vehicle solution or 10 μM synaptamide for 2 days in DMEM/F12 (1:1) containing 10% FBS.

### 2.7. ELISA

To determine the NGF, NMDAR1, NMDAR2A, and ASAHL protein concentration within the hippocampus (ipsi- and contralateral) and cultured astrocyte, the enzyme-linked immunosorbent assay was used. Tissue was harvested 14 days after CCI. The rats (*n* = 7) were anesthetized with 4.5% isofluorane using a rodent anesthesia vaporizer (VetFloTM, Kent Scientific Corporation, Torrington, CT, USA); the hippocampus was quickly extracted and frozen in liquid nitrogen. The primary antibodies used were: recombinant anti-NGF antibody (1:1000, ab52918), anti-NMDAR1 antibody (1:1000, ab52177), anti-NMDAR2A antibody (1:1000, ab203197), all from Abcam (Cambridge, UK), and anti-ASAHL antibody (1:1000, SC-100470, Santa Cruz, Dallas, TX, USA).

Further steps of the ELISA were carried out in accordance with the protocol described in our previous study [17]. Each sample was analyzed twice, and the results were averaged.

### 2.8. Calcium Imaging

Astroglial cells were seeded on a 96 well plate (5 × 10^3^ cells/well) in DMEM, 10% FBS and cultured under standard conditions. In 2 h, synaptamide was added at a concentration of 10 μM. After 48 h, the medium was removed; then, the cells were washed twice with HBSS. After washing, the cells were incubated with 2 μM Fluo-4 AM 494/506 nm (M14206, Thermo Fisher, Waltham, MA, USA) in HBSS for 20 min at 37 °C. After this, the cells were washed twice with HBSS, and then the wells were filled with HBSS. Fluorescence was detected using an inverted confocal microscope (LSM 780, Carl Zeiss, Oberkochen, Germany). Frames were recorded every 800 ms. After 10 s of scanning, 10 μL of 1 mM aspartate solution (aspartic acid sodium salt, A6683, Sigma Aldrich, Saint Louis, MN, USA) diluted in HBSS was added to the well (the primary stock solution was prepared by dilution in 1N NaOH). Control cells were treated under the same conditions with the vehicle. Results were expressed as F/F0, where F is the fluorescence at each time point and F0 is the average baseline fluorescence that was tracked at the start of each experiment. The concentration was calculated according to Grinkevich: C = Kd + F-Fmin/Fmax-F, using software for confocal microscope LSM 780 (Carl Zeiss, Oberkochen, Germany). Calcium concentration was expressed in nmol.

### 2.9. Quantitation of N-Acylethanolamines

For quantitative analysis of the N-acylethanolamines (NAE) content within the brain, 20 rats were used (*n* = 5). The animals were anesthetized with 4.5% isoflurane. The brain was quickly removed, frozen in liquid nitrogen, and stored at −70 °C. An internal standard 22: 0-NAE (0.1 nmol) was added to the frozen rat brain, followed by lipid extraction according to the method of Bligh and Dyer (1959) [25]. LCMS NAE analysis was performed on an Ascentis C18 analytical column (2.1 mm × 100 mm × 3 μm, Supelco, Bellefonte, PA, USA) and LC-MS 8060 (Shimadzu, Kyoto, Japan). The molecular ion and fragment were measured for each compound: 348 → 62 for AEA, 372 → 62 for DHEA, 300 → 62 for PEA, and 384 → 62 for internal standard (22: 0-NAE). Comparison of the peak area with the peak area of the internal standard was used to quantify each NAE in the brain sample. Analysis was performed using LabSolution software (Shimadzu, Kyoto, Japan).

### 2.10. Statistical Data Processing

Mann–Whitney tests and one-way ANOVA tests, followed by post hoc Tukey’s multiple comparison tests, were used for statistical analysis. The data were shown as mean ± SEM, and *p* < 0.05, *p* < 0.01, *p* < 0.001 were considered as statistically significant. GraphPad Prism 4.00 software (GraphPad Software, San Diego, CA, USA) was used for statistical analysis.

## 3. Results

### 3.1. Behavioral Studies

**Figure 2:**
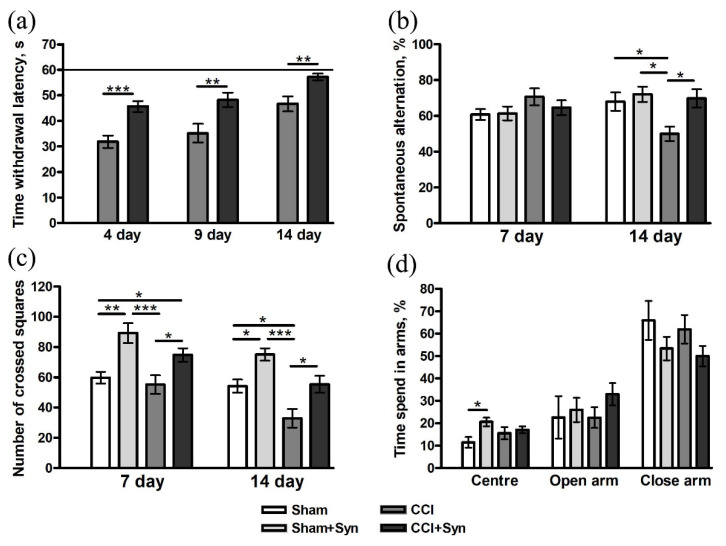
Behavioral effects of CCI and synaptamide treatment. (**a**) Dynamics of the latency period for reaction to thermal stimulus. (**b**) Spontaneous alternation rate differences in Y-maze testing. (**c**) Locomotor activity: the number of crossed squares in the open-field test. (**d**) Anxiety-like behavior testing with the elevated plus-maze (EPM). Data are presented as mean ± SEM, *n* = 19/group, * *p* < 0.05, ** *p* < 0.01, and *** *p* < 0.001 (one-way ANOVA, post-test Tukey).

The moment the hind paw was lifted over the cold plate was recorded to assess cold allodynia. The measurements were taken on the 4th, 9th, and 14th days after the surgery. There was a complete absence of cold allodynia in the Sham and Sham + Syn groups. In the “CCI” and “CCI + Syn” groups, a decrease in the contact time of the limb with the plate was observed. On the 4th day after the surgery, an increase in cold hypersensitivity was revealed in the CCI group (hind paw lift by 31.8 ± 2.4 s), while in the CCI + Syn group, cold allodynia was significantly lower (45.6 ± 2.1 s). A similar picture was observed on the 9th day: the hind paw was raised by 35.1 ± 3.6 s in the “CCI” group and by 48.2 ± 2.8 s in the “CCI + Syn” group. However, we observed a decrease in cold allodynia in the “CCI” group to 46.7 ± 2.9 s by the 14th day of the experiment. At the same time, by the end of the experiment, there was no significant difference in values between the “Sham”, “Sham + Syn”, and “CCI + Syn” groups (Figure 2a).

#### 3.1.1. Working Memory

No impairment of working memory was observed on the 7th day after the surgery in animals from all experimental groups. At the same time, the Y-maze test showed a decrease in working memory in rats of the “CCI” group (50 ± 3.9%) by the 14th day of observation. In synaptamide-treated animals with CCI (69.7 ± 5%), the indicators of working memory did not differ significantly from the “Sham” (68 ± 5.1%) and “Sham + Syn” groups (72 ± 4.2%) throughout the experiment (Figure 2b).

#### 3.1.2. Locomotor Activity

An open-field study revealed that synaptamide treatment significantly induced locomotor activity in CCI- and sham-operated animals 7 days after the surgery. On day 14, a decrease in motor activity in the “CCI” group (33 ± 6.1) was observed in comparison to the “Sham” (54.2 ± 4.3) and “CCI + Syn” (55.4 ± 5.6) groups. At the same time, locomotor activity in the “Sham + Syn” group (75.1 ± 4.1) still significantly exceeded the activity in other groups (Figure 2c).

#### 3.1.3. Anxiety

A study in the elevated plus-maze (EPM) did not reveal significant differences between any of the experimental groups (Figure 2d).

#### 3.1.4. Long-Term Memory

Passive avoidance test did not reveal impairment of long-term memory (24 h) in animals after CCI and synaptamide treatment.

### 3.2. Synaptamide Prevents the Impairment of Neurogenesis after CCI

**Figure 3:**
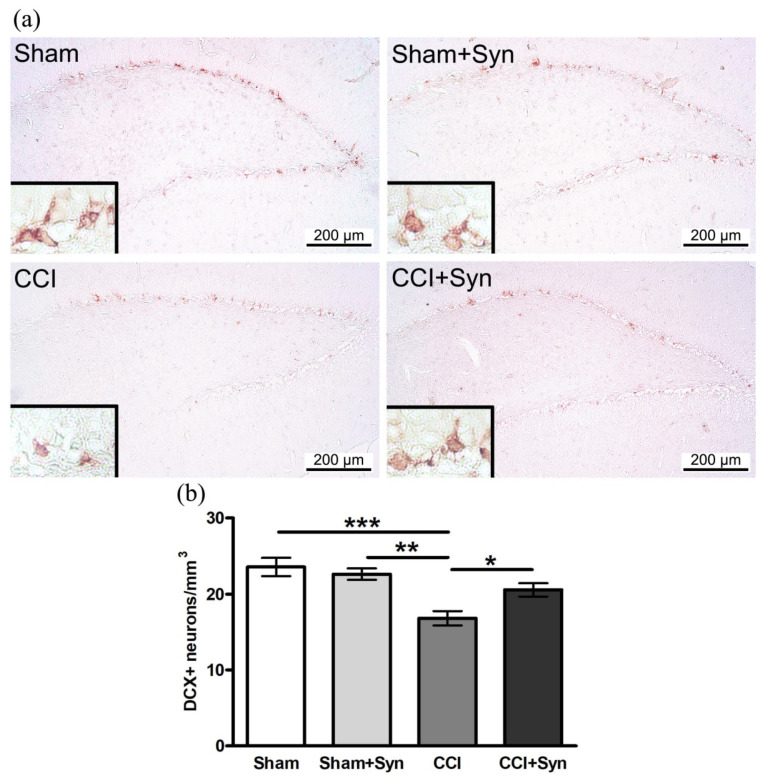
Effect of CCI and synaptamide treatment on hippocampal neurogenesis. (**a**) Representative images of DCX-positive staining. (**b**) Histogram showing the changes in the number of immature neurons (by DCX) in the DG SGZ. Data are presented as mean ± SEM, *n* = 7/group, * *p* < 0.05, ** *p* < 0.01 and *** *p* < 0.001 (one-way ANOVA, post-test Tukey).

DCX is a specific marker for newly generated neurons, which can be used to analyze adult mammalian neurogenesis in the central nervous system [26]. In the neuropathic pain condition, a decrease in neurogenesis in the dentate gyrus subgranular zone of the hippocampus (16.8 ± 0.9 neurons/mm^3^) was observed by the 14th day post-surgery. Synaptamide administration promotes the restoration of a normal level of hippocampal neurogenesis, the number of DCX-positive neurons (20.6 ± 0.9 neurons/mm^3^) in “CCI + Syn” did not significantly differ from the number in the “Sham” (23.6 ± 1.2 neurons/mm^3^) and “Sham + Syn” (22.6 ± 0.7 neurons/mm^3^) groups (Figure 3).

### 3.3. Synaptamide Stabilizes Astrogliosis after CCI

**Figure 4:**
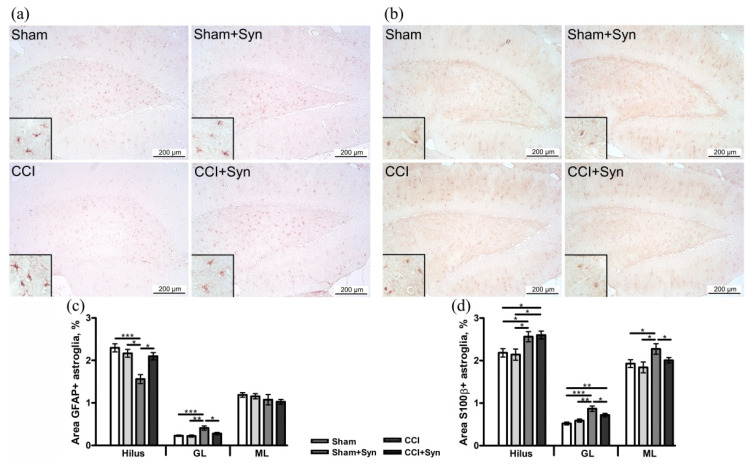
Effect of CCI and synaptamide treatment on hippocampal astroglial activity. Representative images of (**a**) GFAP and (**b**) S100β immunostaining. Histogram demonstrating the % of (**c**) GFAP- and (**d**) S100β-positive staining within the DG regions after the surgery and synaptamide treatment. Data are presented as mean ± SEM, *n* = 7/group, * *p* < 0.05, ** *p* < 0.01 and *** *p* < 0.001 (one-way ANOVA, post-test Tukey).

Astrogliosis is a typical astrocyte response to peripheral nerve injury, accompanied by upregulation of glial fibrillary acidic protein (GFAP) and S100β protein. Peripheral nerve injury and the treatment by analgesic drugs are followed by dynamic alterations in the number and morphology of astrocytes [27]. Evaluation of the GFAP-positive immunostaining showed decreased astroglial activity in the hilus (1.6 ± 0.1%) and increased staining in the molecular layer (0.4 ± 0.04%) of the dentate gyrus of the hippocampus in the “CCI” group compared to “Sham” (2.3 ± 0.1% in the hilus and 0.2 ± 0.01% in the molecular layer) and “Sham + Syn” (2.1 ± 0.1% in the hilus and 0.2 ± 0.02% in the molecular layer). The administration of synaptamide stabilized astrogliosis to the level of sham-operated animals (2.1 ± 0.08% in the hilus and 0.3 ± 0.02% in the molecular layer). In all experimental groups, there was no difference in the activity of astroglia in the granule cell layer of the dentate gyrus of the hippocampus (Figure 4a,c).

Moreover, an increase in S100β protein production, which is expressed mainly by mature astroglia, was observed in all layers of the dentate gyrus of the hippocampus (2.6 ± 0.1% in the hilus, 0.9 ± 0.06% in the granule cell layer, and 2.3 ± 0.1% in the molecular layer) in neuropathic pain condition, in comparison to “Sham” (2.1 ± 0.1% in the hilus, 0.5 ± 0.03% in the granule cell layer, and 1.9 ± 0.1% in the molecular layer) and “Sham + Syn” (2.1 ± 0.1% in the hilus, 0.6 ± 0.03% in the granule cell layer, and 1.8 ± 0.1% in the molecular layer) groups. Synaptamide inhibited astrogliosis in the granule cell layer (0.7 ± 0.04%) and molecular layer (2 ± 0.06%) of the dentate gyrus of the hippocampus, compared to “CCI” group. However, synaptamide administration did not significantly influence astroglial activity in the hilus of the dentate gyrus (2.6 ± 0.1%) in neuropathic pain development (Figure 4b,d).

### 3.4. CCI Influences the Activity of NMDAR1 in the Hippocampus

**Figure 5:**
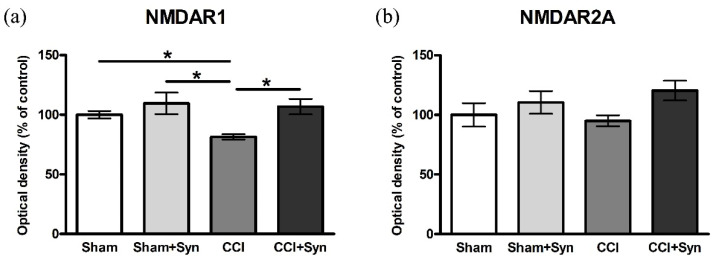
In vivo effect of synaptamide on NMDA receptor in CCI condition. Level of expression of the two subunits of NMDA receptor in the hippocampus: (**a**) NMDAR1 and (**b**) NMDAR2A. Data are presented as mean ± SEM, *n* = 7/group, * *p* < 0.05 (one-way ANOVA, post-test Tukey).

N-methyl-D-aspartate receptor (NMDA) is a type of ligand-gated nonselective ionotropic glutamate receptor, which is highly expressed in the hippocampus and the cerebral cortex. NMDA receptors play an important role in learning and are essential for spatial memory under normal physiological and pathological conditions [28]. In the present study, we show the expression of the two subunits of NMDA receptors (NMDAR1 and NMDAR2A) using the ELISA method. The control values in the “Sham” group were taken as 100%. In neuropathic pain condition, a decrease in NMDAR1 expression was observed (81.4 ± 2.2%); however, synaptamide treatment (106.8 ± 6.4%) maintained the NMDAR1 expression at the control level. In the “Sham + Syn” group (109.6 ± 9%) NMDAR1 expression did not significantly differ from the values of the “Sham” group (Figure 5a). The evaluation of NMDAR2A expression did not reveal significant differences between any of the experimental groups (Figure 5b).

### 3.5. Synaptamide Increases the Ca^2+^ Concentration in Cultured Astrocytes

**Figure 6:**
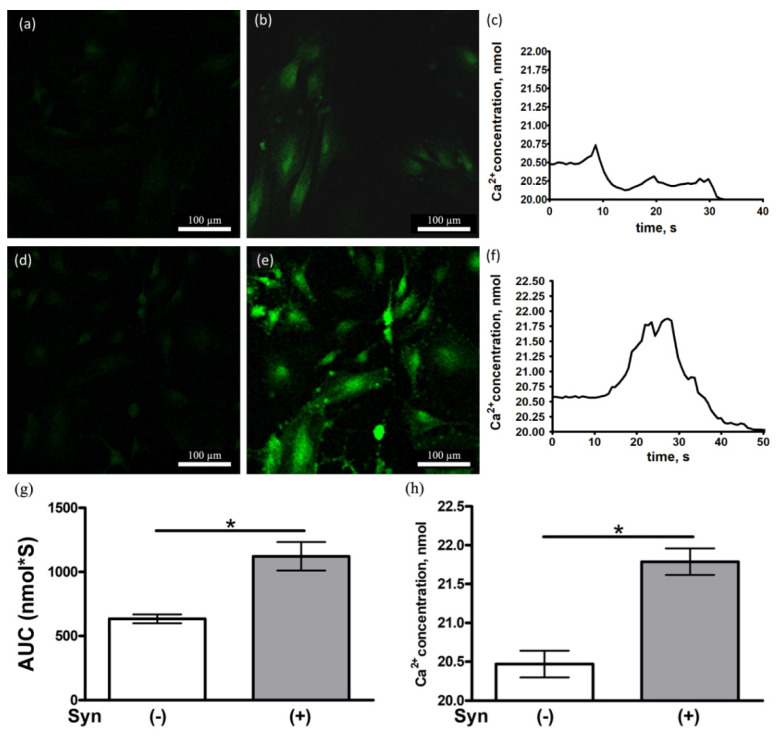
Ca^2+^ level in astrocytes under the influence of aspartate. Change in fluorescence of cells stained with Fluo-4 AM in the group Syn (–) (**a**) 0.8 s, (**b**) 9 s; and Syn (+) (**d**) 0.8 s, (**e**) 28 s. Dynamic ranges of Ca^2+^ concentration increasing in astrocytes in group (**c**) Syn (−) and (**f**) Syn (+). (**g**) AUC (area under the curve) concentration of Ca^2+^ in astrocytes. (**h**) Ca^2+^ concentration at the highest fluorescence peak point. Data are presented as mean ± SEM, *n* = 9/number of analyzed samples, * *p* < 0.05 (Mann–Whitney test).

An aspartate-induced Ca^2+^ influx into astrocytes stained with Fluo-4 caused a parabolic time-dependent fluorescence change (Figure 6a–f). Incubation with 10 µM synaptamide for 48 h increased the Ca^2+^ concentration in aspartate-induced astrocytes, compared to controls (Figure 6g,h). That may be a consequence of an increase in the amount of NMDAR on the surface of astrocyte membranes.

### 3.6. Synaptamide Increases the Activity of Nerve Growth Factor (NGF) In Vivo and In Vitro

Neurotrophins are a family of growth factors that induce growth, differentiation, and survival of neurons in development and pathological conditions [29]. One of the most important neurotrophic factors is the nerve growth factor (NGF), which promotes the outgrowth of neurites and stimulates the differentiation of neurons. In the brain, the highest expression level of NGF mRNA was observed in the hippocampus [30].

**Figure 7:**
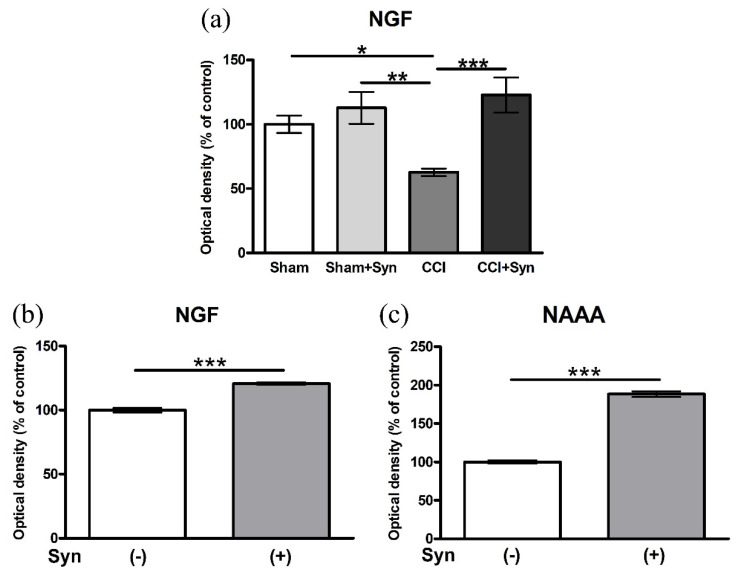
(**a**) Level of NGF expression in the hippocampus after CCI and synaptamide treatment. Data are presented as mean ± SEM, *n* = 7/group, * *p* < 0.05, ** *p* < 0.01, and *** *p* < 0.001 (one-way ANOVA, post-test Tukey). Level of (**b**) NGF and (**c**) NAAA expression in the astroglial cells treated with synaptamide (10 μM). Data are presented as mean ± SEM, *n* = 9/number of analyzed samples, *** *p* < 0.001 (Mann–Whitney test).

The ELISA showed the expression of NGF in the hippocampus of animals with CCI and synaptamide therapy, as well as the expression of NGF in astroglial cell culture after treatment. The control values in the “Sham” group and Syn (−) were taken as 100%. In the “CCI” group, a decrease in NGF expression (62.6 ± 2.9%) was shown, whereas the administration of synaptamide (122.8 ± 13.5%) not only brought the values closer to the level of the “Sham” group, but also tended to increase the NGF activity. In the “Sham + Syn” group (112.8 ± 12.4%), the expression of NGF did not significantly differ from the “Sham” group (Figure 7a). In addition, changes in NGF activity were assessed in synaptamide-treated (10 μM) primary astroglia culture. Synaptamide significantly increased NGF expression up to 120.7 ± 0.9%, compared to the control (Figure 7b). An increase in the expression of the NAAA enzyme (N-acylethanolamine-hydrolyzing acid amidase) up to 188.4 ± 3.7%, in comparison to untreated cells, was shown as evidence of metabolic degradation of synaptamide in astroglia culture (Figure 7c).

### 3.7. N-Acylethanolamines Composition in Plasma and Brain after CCI and Synaptamide Treatment

**Figure 8:**
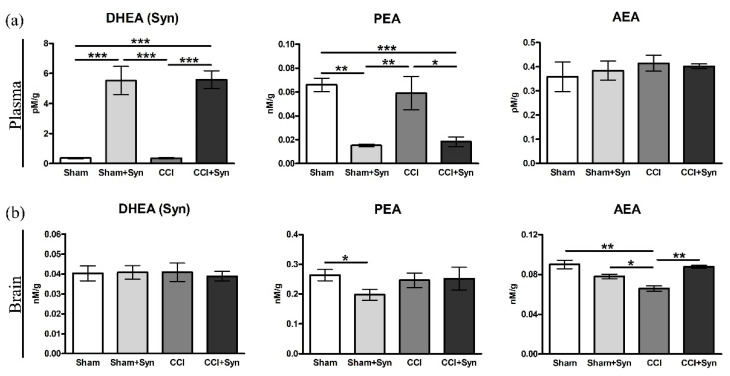
The results of the rat (**a**) brain and (**b**) plasma LC-MS N-acylethanolamines analysis after CCI and synaptamide treatment. Data are presented as mean ± SEM, *n* = 5/group, * *p* < 0.05, ** *p* < 0.01, and *** *p* < 0.001 (one-way ANOVA, post-test Tukey).

Lipid analysis revealed an increase in N-docosahexaenoylethanolamine (DHEA, synaptamide) and a decrease in palmitoylethanolamide (PEA) concentrations in the plasma of synaptamide-treated animals both with neurotrauma (5.6 ± 0.6 pM/g for DHEA and 0.02 ± 0.004 nM/g for PEA) and sham-operated (5.5 ± 0.9 pM/g for DHEA and 0.01 ± 0.001 nM/g for PEA), in comparison to the animals of the “Sham” (0.4 ± 0.04 pM/g for DHEA and 0.06 ± 0.005 nM/g for PEA) and “CCI” (0.4 ± 0.02 pM/g for DHEA and 0.06 ± 0.01 nM/g for PEA) groups. However, there was no increase in arachidonylethanolamide (AEA) concentration in plasma in any of the experimental groups (Figure 8a). At the same time, a decrease in AEA concentration (0.06 ± 0.002 nM/g) was observed in the brain of the animals of the “CCI” group, compared to the “Sham” group (0.09 ± 0.004 nM/g) and “Sham + Syn” group (0.08 ± 0.002 nM/g). Moreover, in the “CCI + Syn” group, the concentration of DHEA (0.09 ± 0.001 nM/g) increased to the level of the sham-operated animals. Interestingly, administration of synaptamide to rats for 14 days did not increase its concentration, nor did it alter PEA levels in the brain (Figure 8b).

## 4. Discussion

The present study aims to assess the effect of synaptamide on the development of neuropathic pain, hippocampal-dependent memory, locomotor activity, astrogliosis, expression of the NMDA receptor, nerve growth factor, and hippocampal neurogenesis in the rat CCI model. Sciatic nerve injury leads to the manifestation of neuropathic pain, as evidenced by the development of cold allodynia from days 4 to 14 after surgery. Synaptamide administration after CCI helps to reduce cold allodynia throughout the observation period, and by the 14th day, this symptom does not manifest in the “CCI + Syn” group. Moreover, the development of neuropathic pain by day 14 is accompanied by a decrease in motor activity. At the same time, synaptamide not only promotes the restoration of locomotor activity in the “CCI + Syn” group but also its significant increase in the “Sham + Syn” group in comparison to the “Sham” group. In addition, behavioral testing has revealed hippocampal-dependent memory impairment by day 14 post-surgery in rats with sciatic nerve injury. Synaptamide treatment prevents the impairment of working spatial memory throughout the observation period. Previous studies have shown that synaptamide prevents sensory and behavioral changes caused by the development of neuropathic pain, such as thermal hyperalgesia, increased anxiety, impaired motor activity, working, and long-term memory [6,31]. However, previously, behavioral aspects of neuropathic pain conditions were examined for 5 weeks after surgery. In the present study, neuropathic pain and administration of synaptamide have not significantly affected long-term memory and anxiety by day 14 of the experiment. Further immunohistochemical and biochemical studies have revealed some mechanisms of the analgesic and neuroprotective effects of synaptamide.

The results of lipid analysis of the blood plasma of the experimental animals confirm the high bioavailability of synaptamide in the case of subcutaneous administration (4 mg/kg/day). Synaptamide treatment for 14 days leads to a fourteen-fold increase in its plasma concentration, in comparison to the vehicle-treated animals. In addition, lipid analysis revealed a significant decrease in the concentration of AEA in the rat brain after CCI. Synaptamide administration helps to maintain the concentration of AEA in the brain at the level of the sham-operated animals, which is probably responsible for the analgesic and cognitive effects of the drug. At the same time, synaptamide treatment for 14 days does not result in a significant accumulation of synaptamide in the brain. The results of our study show that in this case, synaptamide plays the role of a precursor for the synthesis of AEA, since the bulk of synaptamide entering the body is hydrolyzed to DHA and ethanolamine by fatty acid amide hydrolase (FAAH) and NAAA [32,33]. In particular, in our study, a twofold increase in NAAA activity is observed when synaptamide has been added to an astroglial cell culture. In addition, increasing the concentration of synaptamide in the body can inhibit the activity of FAAH and NAAA towards other NAEs due to competitive inhibition [34]. For example, it has previously been shown that synaptamide administration leads to the inhibition of AEA hydrolysis, although to a lesser extent than in the case of the administration of AEA itself [35]. In our previous study, we showed a similar activity of synaptamide; however, when it is administered for 5 weeks, an increase in the concentration of not only AEA, but also other N-acylethanolamines(PEA and oleoylethanolamide (OEA)) s observed [6].

In the present study, cognitive and affective behavioral deficits caused by the development of neuropathic pain are accompanied by impaired hippocampal neurogenesis. Synaptamide administration for 14 days promotes the maintaining of neurogenesis in the DG SZ at the level of sham-operated animals. Previously, synaptamide has been shown to act as an endogenous ligand for the G-protein-coupled receptor 110 (GPR110; ADGRF1) and to induce neuritogenesis and synaptogenesis in hippocampal and cerebral cortex neurons, as well as neuronal differentiation in neural stem cells [12].

NMDA receptors play an important role in learning and memory and are essential for the functioning of working spatial memory [36]. NMDA receptor-mediated synaptic plasticity and its hypofunction are suggested to play a substantial role in a variety of cognitive impairments [37]. The results of our study also indicate that impaired working memory in animals with pain conditions is accompanied by a decrease in the expression of NMDAR1, but not NMDAR2A, a subunit of the NMDA receptor in the hippocampus. Synaptamide administration to animals with CCI promotes the stabilization of the expression of NMDAR1 at the level of sham-operated animals, which is supposedly crucial for improving cognitive functions. Hydrolysis of synaptamide in the body with the formation of DHA and ethanolamine is one of the possible mechanisms of action of synaptamide on the expression of the NMDA receptor. Previously, it has been shown that DHA enhances the NMDA-induced response and simultaneously decreases the kainate-induced response in pyramidal neurons while maintaining the potential at 60 mV under limited voltage conditions. At the same time, this process is not affected by the addition of cyclooxygenase, lipoxygenase, or phospholipase A2 inhibitors. Thus, the authors suggest that DHA can directly target the NMDA receptor, as well as change the composition of membrane lipids and, therefore, increase the activity of the NMDA receptor [38]. Moreover, in our study using calcium imaging, it was shown that the incubation of astrocytes with synaptamide for 48 h increases the NMDA-mediated excitability of cells. This probably indicates an increase in the activity and/or the number of NMDA receptors on the membrane of astroglial cells. In addition, astrocytes and microglia may regulate the functioning of the NMDA receptor on neurons providing additional potential mechanisms for altering NMDA receptor functions in neuropathic pain conditions. Astrocytes are the main source of D-serine, an endogenous co-agonist of the NMDA receptor that interacts with the glycine binding site [39]. The loss of D-serine is observed in the hippocampus and cerebral cortex of rats in aging and stress conditions [40]. In addition, immunohistochemical staining of rat brain sections in conjunction with electron microscopic analysis demonstrated the expression of NMDAR1 and NMDAR2A/B in astrocytes [41]. Activation of NMDA receptors in astrocyte culture leads to an increase in Ca^2+^ signals, both in processes and in cell somas [42].

Astrocytes are the most important component of the trophic, reparative, and homeostatic systems of the central nervous system. This type of cell reacts to various pathological conditions, such as trauma, infection, ischemia, and stress, by going into the activated state (astrogliosis) [43]. The development of neuropathic pain after peripheral nerve injury observed in our study is accompanied by the diverse activity of GFAP- and S100β-positive astroglia. By the 14th day after surgery, the activity of GFAP-positive astroglia is decreased in the hilus of the dentate gyrus of the hippocampus, while the activity of S100β-positive astroglia is increased in all regions of the dentate gyrus of the hippocampus. It is known that GFAP is expressed in all types of astrocytes in the brain, including mature and immature astrocytes, while S100β marks only mature astrocytes [44]. Therefore, it can be assumed that the astrogliosis observed in our study occurs due to the migration and maturation of astroglia, but not due to proliferation processes. Synaptamide administration stabilizes astrogliosis at the level of sham-operated animals in almost all layers of the hippocampal dentate gyrus. Previously, it has been shown that synaptamide normally does not affect the expression of the GFAP protein in the hippocampus of experimental animals [18]. Therefore, we hypothesize that synaptamide may reduce astrogliosis in the hippocampus by inhibiting the activity of microglial cells and the release of pro-inflammatory cytokines, which has been shown in our previous study [6]. The development of astrogliosis mediated by the activity of proinflammatory microglia in neuropathic pain condition has been demonstrated in several studies [45]. Moreover, synaptamide affects the functional state of hippocampal astroglia by enhancing the expression of neurotrophins, including NGF, which were downregulated in 14 days after CCI, as demonstrated in the present study. At the same time, ELISA has shown that the treatment of astroglial cells with synaptamide (10 μM) leads to an NGF expression increase. As previously shown, NGF can affect astrocytes by activating p75 signaling in an autocrine manner [46]. The new properties of the p75 receptor that have been described indicate its interaction with various receptors and adapter proteins [47]. Through p75 signaling, NGF promotes the inhibition of astrocyte activation [46]. At the same time, synaptamide promotes an increase in NGF expression by enhancing cAMP/PKA signaling when interacting with the GPR110 receptor. The relationship between the expression of neurotrophins, including NGF, and an increase in the activity of the cAMP/PKA signaling pathway has been previously shown [48]. Ultimately, the regulation of astrogliosis and NGF production in the hippocampus is likely to contribute to the normalization of cognitive functions and neurogenesis in neuropathic pain conditions.

## 5. Conclusions

The results of this study demonstrate that synaptamide has a complex effect on the development of neuropathic pain after CCI, which indicates its high therapeutic potential. Further detailed study of the neuroprotective mechanisms of synaptamide is necessary for the introduction of the drug into clinical practice for the treatment and prevention of nervous system disorders.

## Data Availability

The data are available from the corresponding author upon reasonable request.
